# Ventilatory Efficiency in Children and Adolescents Born Extremely Preterm

**DOI:** 10.3389/fphys.2017.00499

**Published:** 2017-07-13

**Authors:** Julie Hestnes, Hedda Hoel, Ole J. Risa, Hanna O. Romstøl, Ola Røksund, Bente Frisk, Einar Thorsen, Thomas Halvorsen, Hege H. Clemm

**Affiliations:** ^1^Department of Clinical Science, University of Bergen Bergen, Norway; ^2^Department of Occupational Therapy, Physiotherapy and Radiography, Western Norway University of Applied Science Bergen, Norway; ^3^Department of Physiotherapy, Haukeland University Hospital Bergen, Norway; ^4^Department of Paediatrics, Haukeland University Hospital Bergen, Norway

**Keywords:** extremely preterm born, exercise, bronchopulmonary dysplasia, respiratory mechanics, pulmonary gas exchange, breathing pattern

## Abstract

**Purpose:** Children and adolescents born extremely preterm (EP) have lower dynamic lung volumes and gas transfer capacity than subjects born at term. Most studies also report lower aerobic capacity. We hypothesized that ventilatory efficiency was poorer and that breathing patterns differed in EP−born compared to term−born individuals.

**Methods:** Two area−based cohorts of participants born with gestational age ≤28 weeks or birth weight ≤1000 g in 1982−85 (*n* = 46) and 1991–92 (*n* = 35) were compared with individually matched controls born at term. Mean ages were 18 and 10 years, respectively. The participants performed an incremental treadmill exercise test to peak oxygen uptake with data averaged over 20 s intervals. For each participant, the relationship between exhaled minute ventilation ($\dot{\text{V}}$_E_) and carbon dioxide output ($\dot{\text{V}}$CO_2_) was described by a linear model, and the relationship between tidal volume (V_T_) and $\dot{\text{V}}$_E_ by a quadratic model. Multivariate regression analyses were done with curve parameters as dependent variables, and the categories EP vs. term−born, sex, age, height, weight and forced expiratory volume in 1 s (FEV_1_) as independent variables.

**Results:** In adjusted analyses, the slope of the $\dot{\text{V}}$_E_−$\dot{\text{V}}$CO_2_ relationship was significantly steeper in the EP than the term-born group, whereas no group difference was observed for the breathing pattern, which was related to FEV_1_ only.

**Conclusion:** EP-born participants breathed with higher $\dot{\text{V}}$_E_ for any given CO_2_ output, indicating lower ventilatory efficiency, possibly contributing to lower aerobic capacity. The breathing patterns did not differ between the EP and term−born groups when adjusted for FEV_1_.

## Introduction

Immense progress during the past 30–40 years in the care of infants born extremely preterm (EP) has markedly increased their prospects of long-term survival. While in the 1970 s most of these infants died, the situation today is reversed with overall survival rates in the range of 80–90% (Markestad et al., 2005). When born at this early stage, gas exchange must take place in developmentally fetal lungs still in their canalicular or saccular phase, disturbing growth and development (Jobe and Bancalari, 2001). Studies of EP−born children and adolescents report altered acinar structures with fewer and larger alveoli and lower alveolar capillary density, lower dynamic lung volumes, and lower diffusing capacity for carbon monoxide (Cutz and Chiasson, 2008; Satrell et al., 2013; Chang et al., 2016). Most studies on EP–born children and adolescents find lower peak oxygen uptake ($\dot{\text{V}}$O_2peak_) (Kilbride et al., 2003; Smith et al., 2008; Burns et al., 2009; Welsh et al., 2010; Clemm et al., 2012, 2014, 2015).

Ventilatory mechanical limitations, including limited expansion of the tidal volume (V_T_), or alveolar gas exchange limitations, may contribute to lower peak exercise capacity. MacLean et al. (2016) reported in a recent study that the slope of the relationship between minute ventilation ($\dot{\text{V}}$_E_) and carbon dioxide output ($\dot{\text{V}}$CO_2_) was steeper in children born EP than in a term−born control group. They also found lower peak minute ventilation ($\dot{\text{V}}$_Epeak_) and peak tidal volume (V_Tpeak_). The increased slope of the $\dot{\text{V}}$_E_−$\dot{\text{V}}$CO_2_ relationship indicate increased dead space ventilation which could be related to increased alveolar dead space, or increased anatomical dead space ventilation due to differences in breathing pattern with a higher breathing frequency (B_f_). Cardiovascular effects linked to developmental abnormalities or secondary to the changes in pulmonary function may also contribute to a lower $\dot{\text{V}}$O_2peak_. Reduced alveolar capillary capacitance and reduced conductance of the pulmonary circulation could contribute to a reduction in maximal cardiac output and exercise induced pulmonary hypertension (Abman et al., 2017).

Evaluation of exercise capacity by maximal or peak responses cannot provide information about response trajectories from rest to peak. Despite standards for achieving a maximum $\dot{\text{V}}$O_2_, such end−point criteria are often not met in children (Armstrong and Welsman, 2007). The relationships between $\dot{\text{V}}$_E_ and $\dot{\text{V}}$CO_2_ up to the isocapnic compensation point and between heart rate (HR) and $\dot{\text{V}}$O_2_ are known to be linear, and we have previously shown that there is a quadratic relationship between V_T_ and $\dot{\text{V}}$_E_ (Frisk et al., 2014). When comparing the trajectories by the curve parameters of the responses, the error related to whether a maximum response was achieved or not is reduced.

In the present study, we have compared the $\dot{\text{V}}$_E_ vs. $\dot{\text{V}}$CO_2_, HR vs. $\dot{\text{V}}$O_2_ and V_T_ vs. $\dot{\text{V}}$_E_ relationships during progressively incremental treadmill exercise in EP and term−born children, and examined possible predictors for the responses including sex, height, weight, age, smoking, leisure−time physical activity, forced vital capacity (FVC) and forced expiratory volume in 1 s (FEV_1_). We hypothesized that the slope of the $\dot{\text{V}}$_E_ − $\dot{\text{V}}$CO_2_ relationship is higher in EP−born subjects, as demonstrated by MacLean et al. (2016), and that breathing pattern would be different if the increased slope is related to increased anatomical dead space ventilation.

## Methods

Two population-based birth-cohorts of participants born at gestational age ≤28 weeks or birth weight ≤1,000 gram in 1982–1985 (*n* = 46) and 1991–1992 (*n* = 35) were compared with individually matched control groups born at term. Mean ages at the examinations were 18 and 10 years, respectively. These two EP-born cohorts have been extensively followed up for decades, and are hereafter referred to as the 1982–1985 and the 1991–1992 cohorts. Details as regards their recruitment have been reported in previous communications (Halvorsen et al., 2006; Clemm et al., 2012). Background information was obtained by a questionnaire, including information about level of leisure−time physical activity and smoking. Parental answers were used for the 1991–1992 cohort. FVC and FEV_1_ were measured with a Vmax22 spirometer (*SensorMedics, Yorba Linda, CA, USA*) in accordance with European Respiratory Society and American Thoracic Society quality criteria (American Thoracic and American College of Chest, 2003).

An incremental maximal treadmill (*ELG 70 Woodway, Weil am Rhein, Germany*) exercise test was done in all participants, using the same computerized modified Bruce protocol. Speed and inclination were gradually increased every 90 s. Oxygen uptake ($\dot{\text{V}}$O_2_) and carbon dioxide output ($\dot{\text{V}}$CO_2_), minute ventilation ($\dot{\text{V}}$_E_), tidal volume (V_T_) and heart rate (HR) were measured breath by breath by a Vmax29 exercise unit (*SensorMedics, Yorba Linda, CA, USA*). The test was stopped when the participants indicated exhaustion. Anthropometric characteristics, lung function data and peak responses to the exercise test are given in Table 1. The acquisition of background information by questionnaires, and the measurements of lung function and cardiopulmonary exercise capacity have been described in detail in a previous paper analyzing the peak responses to exercise (Clemm et al., 2012). In this present paper, which is based on the same data, we provide extended analyses of the exercise responses, including the progress of submaximal responses.

**Table 1:** Participant characteristics and peak responses to progressively incremental exercise test on treadmill.

	**EP-born participants**		**Term-born participants**	
	**Male (*n* = 11)**	**Female (*n* = 20)**	**Male (*n* = 12)**	**Female (*n* = 22)**
**1991–1992 birthcohort (age 10 years)**				
Birth weight (gram)	968 ± 196	913 ± 210	3, 565 ± 292	3, 563 ± 271
GA at birth (weeks)	26 ± 1.3	27 ± 1.9	^**^	^**^
Height (cm)	138 ± 6^*^	140 ± 9	146 ± 7	144 ± 6
Body mass (kg)	31 ± 3^*^	35 ± 12	37 ± 7	39 ± 7
FVC (L)	2.14 ± 0.31^*^	2.11 ± 0.49^*^	2.58 ± 0.23	2.37 ± 0.29
FEV_1_ (L)	1.70 ± 0.21^*^	1.84 ± 0.43^*^	2.26 ± 0.23	2.09 ± 0.29
$\dot{\text{V}}$O_2*peak*_ (L · min ^−1^)	1.45 ± 0.18^*^	1.41 ± 0.23^*^	1.81 ± 0.11	1.68 ± 0.24
$\dot{\text{V}}$CO_2*peak*_ (L · min ^−1^)	1.52 ± 0.23^*^	1.52 ± 0.26^*^	1.97 ± 0.19	1.82 ± 0.32
$\dot{\text{V}}$_*Epeak*_ (L · min ^−1^)	54.9 ± 9.1^*^	52.5 ± 7.6^*^	63.3 ± 8.4	60.7 ± 13.2
HR_*peak*_ (min^−1^)	198 ± 8	197 ± 7	203 ± 7	201 ± 12
	**Male (*****n*** = **20)**	**Female (*****n*** = **17)**	**Male (*****n*** = **25)**	**Female (*****n*** = **20)**
**1982–1985 birth-cohort (age 18 years)**				
Birth weight (gram)	1, 011 ± 189	1, 029 ± 201	3, 423 ± 307	3, 490 ± 338
GA at birth (weeks)	27 ± 1.3	27 ± 1.2	^**^	^**^
Height (cm)	176 ± 6	164 ± 4^*^	177 ± 6	168 ± 6
Body mass (kg)	68 ± 15	59 ± 9	68 ± 8	67 ± 15
FVC (L)	4.79 ± 0.80	3.60 ± 0.46	5.04 ± 0.75	3.96 ± 0.62
FEV_1_ (L)	3.93 ± 0.59^*^	3.03 ± 0.42^*^	4.47 ± 0.68	3.52 ± 0.47
$\dot{\text{V}}$O_2peak_ (L·min^−1^)	3.50 ± 0.62	2.41 ± 0.37	3.79 ± 0.57	2.68 ± 0.47
$\dot{\text{V}}$CO_2peak_ (L·min^−1^)	3.80 ± 0.70	2.63 ± 0.46^*^	4.17 ± 0.64	3.00 ± 0.51
$\dot{\text{V}}$_Epeak_ (L·min^−1^)	109.8 ± 23.9	76.1 ± 16.3	120.2 ± 25.3	82.9 ± 9.8
HR_peak_ (min^−1^)	197 ± 16	193 ± 11	198 ± 9	195 ± 10

*Mean ± 1 standard deviation*.

* *Significantly different from term-born participants of same sex p < 0.05*.

*GA gestational age, FVC forced vital capacity, FEV_1_ forced expired volume in 1 s, $\dot{\text{V}}$O_2peak_ peak oxygen uptake, $\dot{\text{V}}$CO_2peak_ peak carbon dioxide output, $\dot{\text{V}}$_Epeak_ peak minute ventilation, HR_peak_ peak heart rate*.

** GA in term−born participants were > 37 weeks, exact number not applicable

The Regional Committee for Medical and Health Research Ethics of Western Norway approved the studies (REK-Vest 99.2000). Informed written consent was obtained from all participants or from their parents if they were less than 16 years of age, in accordance with the Declaration of Helsinki. Most of the participants, and all the children born in 1991–1992 (EP-born and term-born), had at least one adult guardian present during testing, usually one of the parents.

### Data processing and statistics

Breath by breath measurements of $\dot{\text{V}}$_E_, V_T_, $\dot{\text{V}}$O_2_, $\dot{\text{V}}$CO_2_ and HR were averaged over 20 s intervals. For each participant the relationship between $\dot{\text{V}}$_E_ and $\dot{\text{V}}$CO_2_ was described by a linear regression model up to the isocapnic compensation point; $\dot{\text{V}}$_E_ = a + b·$\dot{\text{V}}$CO_2_. The relationship between HR and $\dot{\text{V}}$O_2_ was also described by a linear model; HR = a + b·$\dot{\text{V}}$O_2_. The relationship between V_T_ and $\dot{\text{V}}$_E_ was described by a quadratic regression model; V_T_ = a + b·$\dot{\text{V}}$_E_ + c·${\dot{\text{V}}}_{\text{E}}^{2}$. The goodness of fit for each response in each participant was evaluated by the F-statistics and the adjusted coefficient of determination (R^2^). *P* < 0.05 was required for inclusion of the participant in further analyses.

Descriptive statistics were used to characterize the study population, including mean and standard deviation (SD), and median and range, as appropriate. Independent samples *t*-tests were used to compare continuous variables. Bivariate and multivariate linear regression models were constructed, using the curve parameters a, b, and c as descriptors of the respective exercise responses (dependent variables), and the categories EP vs. term-born, height, weight, sex, FEV_1_ and birth-cohort (reflecting age) as independent and potentially explanatory variables.

The variables included in the multivariate linear regression models were based on results of univariate analyses. The variables EP vs. term-born, sex and birth-cohort were included *a priori*. FVC was not included in the analyses because of extensive co-linearity with FEV_1_. FEV_1_ was included as absolute values and not as percentages of predicted or z-scores since sex, age and height were included in all models. Body mass index (BMI) was not included as variable since it includes height and weight. Smoking, which was reported by participants in the elder cohort only, and level of habitual physical activity were not related to the dependent variables in univariate analysis.

Estimated regression coefficients are presented with 95% confidence intervals (CI). The significance level was set at 0.05. The data analyses were performed using IBM SPSS Statistics 21 (SPSS Inc., Chicago, IL).

## Results

In a previous study we have demonstrated that the EP-born participants had lower height, body mass, FVC and FEV_1_ than the term-born controls, and their peak responses to exercise were also lower (Clemm et al., 2012).

The relationships $\dot{\text{V}}$_E_ vs. $\dot{\text{V}}$CO_2_ and HR vs. $\dot{\text{V}}$O_2_ could be satisfactorily described by the linear models in all participants. Median *R*^2^ was 0.98 (range 0.96–0.99) and 0.90 (range 0.71–0.99), respectively. The relationship between V_T_ vs. $\dot{\text{V}}$_E_ was satisfactorily described by a quadratic model in all but two participants. Median *R*^2^ was 0.95 (range 0.45–0.99). The curve parameters for the relationships are given in Table 2, split by birth−cohort and sex.

**Table 2:** Curve parameters (a, b, and c) describing the relationships between $\dot{\text{V}}$_E_ and $\dot{\text{V}}$CO_2_, between HR and $\dot{\text{V}}$O_2_ and between V_T_ and $\dot{\text{V}}$_E_.

	**EP-born participants**		**Term-born participants**	
	**Male (*n* = 11)**	**Female (*n* = 20)**	**Male (*n* = 12)**	**Female (*n* = 22)**
**1991–1992 birth-cohort (age 10 years)**				
**$\dot{\text{V}}$**_E_ = **a** + **b**·**$\dot{\text{V}}$CO_2_**				
a	2.4 ± 1.5	2.2 ± 1.4	2.3 ± 1.3	2.1 ± 1.6
b	32.8 ± 2.1^*^	32.1 ± 2.8	29.5 ± 2.2	30.7 ± 3.4
**HR = a + b·$\dot{\text{V}}$O_2_**				
a	75 ± 12	70 ± 10	74 ± 13	77 ± 16
b	93 ± 10^*^	100 ± 15^*^	79 ± 9	82 ± 18
**V_T_ = a + b·$\dot{\text{V}}$_E_ + c·**${\dot{\text{V}}}_{\text{E}}^{2}$				
a	0.17 ± 0.09	0.17 ± 0.10	0.14 ± 0.10	0.22 ± 0.20
b (·10^−2^)	2.4 ± 1.0	2.6 ± 0.6	3.0 ± 0.9	2.8 ± 0.8
c (·10^−4^)	−2.0 ± 1.6	−1.9 ± 1.0	−2.1 ± 1.1	−2.1 ± 1.4
	**Male (*****n*** = **20)**	**Female (*****n*** = **17)**	**Male (*****n*** = **25)**	**Female (*****n*** = **20)**
**1982–1985 birth-cohort (age 18 years)**				
**$\dot{\text{V}}$_E_ = a + b·$\dot{\text{V}}$CO_2_**				
a	2.7 ± 1.7	3.3 ± 1.9	2.3 ± 2.0	4.2 ± 1.6
b	25.4 ± 2.0	26.2 ± 3.9	25.1 ± 2.8	25.2 ± 3.1
**HR = a + b·$\dot{\text{V}}$O_2_**				
a	65 ± 11	68 ± 13	68 ± 12	75 ± 14
b	40 ± 8	57 ± 9	39 ± 5	51 ± 12
**V_T_ = a + b·$\dot{\text{V}}$_E_ + c·**${\dot{\text{V}}}_{\text{E}}^{2}$				
a	0.24 ± 0.20	0.09 ± 0.17	0.29 ± 0.16	0.17 ± 0.16
b (·10^−2^)	3.7 ± 0.9	4.5 ± 1.2	3.8 ± 0.9	4.0 ± 1.1
c (·10^−4^)	−1.8 ± 0.8	−3.5 ± 1.9^*^	−1.7 ± 1.0	−2.4 ± 1.1

*Mean ± 1 standard deviation*.

* significantly different from controls of same sex p < 0.05

*$\dot{\text{V}}$O_2_ oxygen uptake, $\dot{\text{V}}$CO_2_ carbon dioxide output, $\dot{\text{V}}$_E_ minute ventilation, HR heart rate*.

The intercept of the linear relationship $\dot{\text{V}}$_E_ vs. $\dot{\text{V}}$CO_2_ was significantly related to FEV_1_ and cohort, but not to the other independent variables included in the model. Participants in the 1991–1992 birth-cohort and participants with a higher FEV_1_ had a lower intercept (Table 3). The slope was higher in the 1991–1992 birth−cohort, and higher with increasing height, and also higher in the EP than the term−born groups.

**Table 3:** Multiple regression analyses for the curve parameters describing the relationship between $\dot{\text{V}}$_E_ and $\dot{\text{V}}$CO_2_, $\dot{\text{V}}$_E_ = a + b·$\dot{\text{V}}$CO_2_

	**Bivariate**		**Multivariate**			
	**B**	**p**	**B**	**Adjusted B**	**95% CI**	**p**
**Curve Parameter a**						
Case/Control	0.13	0.802	0.37	0.11	−0.22, 0.97	0.213
Sex	0.44	0.130	0.30	0.09	−0.33, 0.94	0.348
FEV_1_	0.12	0.387	−1.17	−0.73	−1.89, −0.45	0.002
Height	0.02	0.026	0.05	0.51	−0.003, 0.11	0.063
Body mass	0.02	0.039	0.001	0.01	−0.03, 0.03	0.941
Cohort	−0.87	0.003	−1.42	−0.40	−2.68, −0.16	0.028
**Curve Parameter b**						
Case/Control	−1.39	0.043	−1.12	−0.14	−2.13, −0.12	0.029
Sex	1.49	0.029	0.15	0.02	−0.93, 1.23	0.782
FEV_1_	−2.42	0.000	1.06	0.28	−0.17, 2.29	0.090
Height	−0.18	0.000	−0.13	−0.51	−0.23, −0.03	0.011
Body mass	−0.15	0.000	−0.03	−0.14	−0.09, 0.02	0.268
Cohort	5.85	0.000	3.04	0.37	0.90, 5.18	0.006

*FEV_1_ forced expired volume in 1 s, $\dot{\text{V}}$CO_2_ carbon dioxide output*.

The intercept of the linear relationship HR vs. $\dot{\text{V}}$O_2_ was significantly related to sex, height, body mass and birth-cohort, but not to EP vs. term-born (Table 4). The slope was related to sex, height, body mass and cohort, and significantly higher in the EP-born group. Birth–cohort (1982-1985 vs. 1991–1992) reflected not only the age differences, but also the fact that participants in the 1991–1992 cohort were pre-pubertal and participants in the 1982–1985 cohort post-pubertal.

**Table 4:** Multiple regression analyses for the curve parameters describing the relationship between HR and $\dot{\text{V}}$O_2_, HR = a + b·$\dot{\text{V}}$O_2_.

	**Bivariate**		**Multivariate**			
	**B**	**p**	**B**	**Adjusted B**	**95% CI**	**p**
**Curve Parameter a**						
Case/Control	4.23	0.051	4.87	0.19	0.37, 9.37	0.034
Sex	3.72	0.087	1.68	0.07	−3.18, 6.53	0.495
FEV_1_	−2.08	0.027	1.48	0.13	−4.01, 6.98	0.597
Height	−0.18	0.005	−0.35	−0.45	−0.78, 0.06	0.113
Body mass	−0.09	0.027	0.03	0.04	−0.21, 0.28	0.803
Cohort	4.17	0.049	−1.82	−0.07	−11.41, 7.76	0.708
**Curve Parameter b**						
Case/Control	−10.5	0.013	−6.24	−0.12	−10.01, −2.47	0.001
Sex	18.2	0.000	6.88	0.14	2.81, 10.94	0.001
FEV_1_	−19.7	0.000	−0.12	−0.01	−4.73, 4.49	0.959
Height	−1.4	0.000	−0.42	−0.28	−0.79, −0.06	0.024
Body mass	−1.2	0.000	−0.38	−0.28	−0.59, −0.18	0.000
Cohort	43.0	0.000	17.23	0.34	9.2, 25.3	0.000

*FEV_1_ forced expired volume in 1 s, HR heart rate, $\dot{\text{V}}$O_2_ oxygen uptake*.

The curve parameters of the quadratic relationship between V_T_ and $\dot{\text{V}}$_E_ were all different between the cohorts, and the curvature (parameter c) was related to FEV_1_ (Table 5). Participants in the 1991−1992 cohort and participants with a lower FEV_1_ had a lower maximal V_T_ at a lower $\dot{\text{V}}$_E._ At a given $\dot{\text{V}}$_E_ there was no difference in V_T_ between the EP and term−born groups when adjusted for FEV_1_.

**Table 5:** Multiple regression analyses for the curve parameters describing the relationship between V_T_ and $\dot{\text{V}}$_E_, V_T_ = a + b·$\dot{\text{V}}$_E_ + c·${\dot{\text{V}}}_{\text{E}}^{2}$

	**Bivariate**		**Multivariate**			
	**B**	**p**	**B**	**Adjusted B**	**95% CI**	**p**
**Curve Parameter a**						
Case/Control	0.05	0.108	0.02	0.05	−0.04, 0.08	0.541
Sex	−0.06	0.025	−0.02	0.03	-0.09, 0.04	0.464
FEV_1_	0.03	0.010	0.05	0.34	−0.02, 0.12	0.157
Height	0.01	0.041	0.002	0.16	−0.01, 0.01	0.570
Body mass	0.01	0.150	−0.001	−0.09	−0.01, 0.01	0.594
Cohort	−0.03	0.381	0.097	0.29	−0.03, 0.22	0.124
**Curve Parameter b (·10^−2^)**						
Case/Control	0.1	0.747	0.1	2.9	−0.3, 0.4	0.701
Sex	1.0	0.915	0.2	8.6	−0.2, 0.6	0.296
FEV_1_	4.0	0.000	−0.1	−12.3	−0.6, 0.3	0.554
Height	0.1	0.000	0.01	6.0	−0.1, 0.1	0.809
Body mass	0.03	0.000	0.03	4.9	−0.1, 0.1	0.750
Cohort	-1.3	0.000	−1.3	−57.2	−2.0, −0.6	0.001
**Curve Parameter c (·10^−4^)**						
Case/Control	0.25	0.265	−0.04	−160	−4.9, 4.1	0.849
Sex	−0.6	0.006	−0.30	−119	−0.8, 1.7	0.197
FEV_1_	0.15	0.135	0.64	524	0.1, 1.2	0.025
Height	0.04	0.570	0.02	280	−0.4, 0.5	0.920
Body mass	0.02	0.782	0.001	40	−0.2, 0.2	0.983
Cohort	0.24	0.282	1.0	566	0.6, 2.5	0.020

*FEV_1_ forced expired volume in 1s, V_T_ tidal volume, $\dot{\text{V}}$_E_ minute ventilation*.

**Figure 1:**
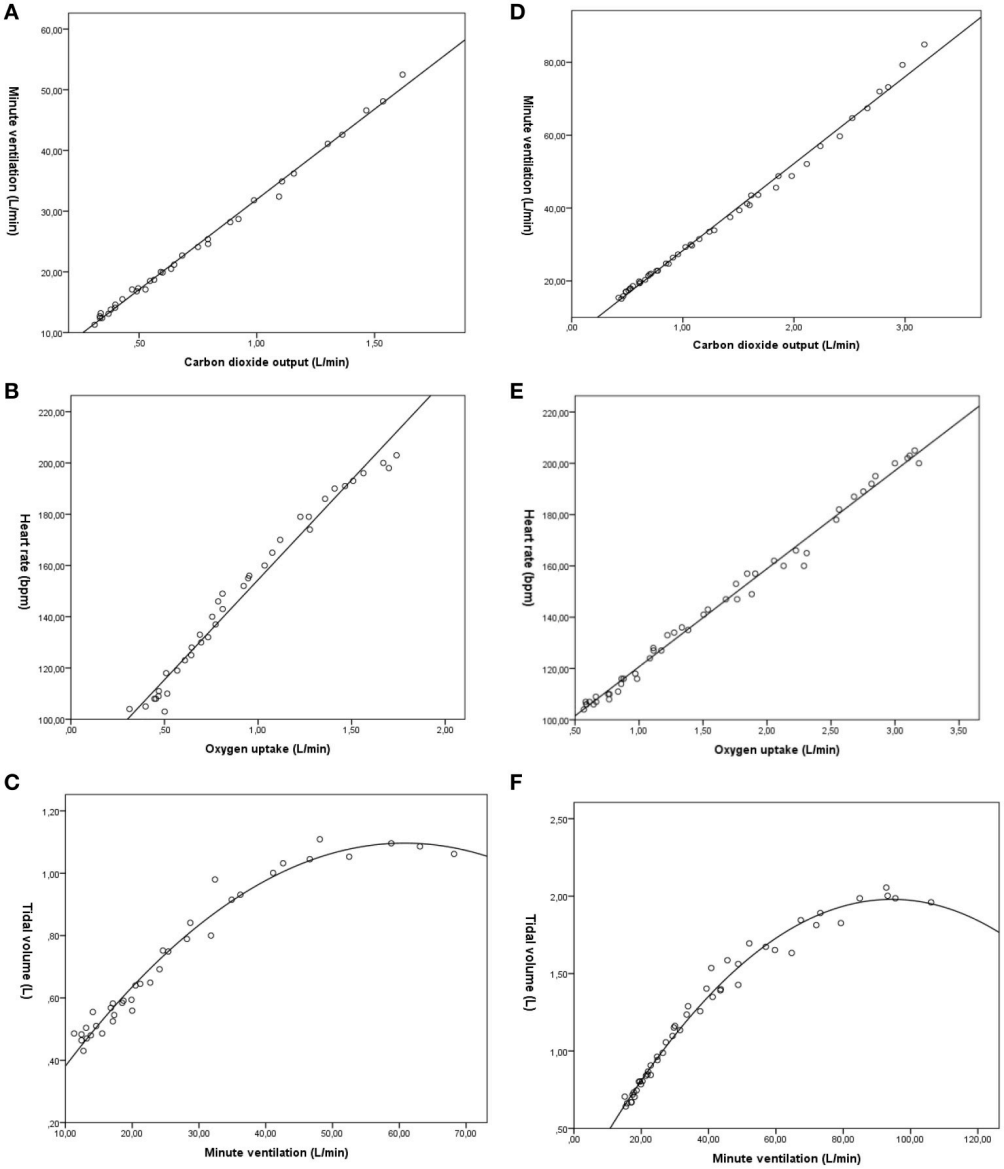
The relationships between ventilation and carbon dioxide output, heart rate and oxygen uptake, and tidal volume and ventilation. Two randomly selected participants, one from the 1991–1992 cohort at 10 years of age (left column **A–C**), and one from the 1982–1985 cohort at 18 years of age (right column **D–F**).

The relationships are demonstrated in Figure 1.

## Discussion

This is the first study to show that cardiopulmonary exercise responses can be satisfactorily described in the vast majority of preterm born children and young adults by using mathematical regression models previously applied and tested only in adult patients with COPD. The EP−born participants differed from the term−born controls in that the slopes for the relationships between $\dot{\text{V}}$E vs. $\dot{\text{V}}$CO_2_, and HR vs. $\dot{\text{V}}$O_2_, were significantly higher; i.e., $\dot{\text{V}}$E was significantly higher for a given CO_2_ output, and HR was higher for a given O_2_ uptake after adjusting for anthropometric characteristics and FEV_1_. The breathing patterns were related to FEV_1_ irrespective of EP or term−born group status.

The higher slope of the $\dot{\text{V}}$_E_−$\dot{\text{V}}$CO_2_ relationship for EP−born indicates higher dead space ventilation. This could be related to increased ventilation of the anatomical dead space because of shallower breathing at higher breathing frequencies (Chang et al., 2016). There were no differences in the relationship between V_T_ and $\dot{\text{V}}$_E_ between the groups after adjusting for FEV_1_. Since $\dot{\text{V}}$_E_ = V_T_· B_f_, participants born extremely preterm will have the same B_f_ at a given $\dot{\text{V}}$_E_, indicating that ventilation of the anatomical dead space is not different after adjusting for FEV_1_. Thus, differences in breathing pattern cannot explain the higher slope of the $\dot{\text{V}}$_E_ − $\dot{\text{V}}$CO_2_ relationship, and differences in alveolar dead space ventilation are more likely. The higher slope means a higher ventilatory equivalent for CO_2_. When the ventilatory equivalent, which is $\dot{\text{V}}$_E_/$\dot{\text{V}}$CO_2_, is related to $\dot{\text{V}}$CO_2_ it is just a mathematical transformation of the $\dot{\text{V}}$_E_ − $\dot{\text{V}}$CO_2_ relationship. A higher alveolar dead space in participants born extremely preterm is consistent with a lower diffusion capacity for carbon monoxide. The infants are born with developmentally fetal lungs, and further development of the lung is disturbed resulting in fewer and larger alveoli, and lower alveolar capillary density. There are few autopsy studies from EP-born individuals after infancy (Cutz and Chiasson, 2008), but those that we have from infants dying from severe bronchopulmonary dysplasia (BPD) suggest dysplastic acini with fewer and larger alveoli, and lower alveolar capillary density (De Paepe et al., 2006; Cutz and Chiasson, 2008), possibly pathways to later increased alveolar deadspace ventilation. Imaging studies generally agree that EP birth is associated with long−standing and structural injuries, although the findings have varied (Aukland et al., 2006; Wong et al., 2008; Simpson et al., 2017). Recent studies suggest that although injured in their neonatal period, alveolar growth might continue in these children, at least until school age, so these issues are not solved (Weibel, 2008; Narayanan et al., 2013).

BPD represents a categorization of preterm born individuals, based on prolonged requirements for supplemental oxygen (Jobe and Bancalari, 2001) and primarily (but not exclusively) reflects the severity of neonatal respiratory disease. As regards EP-born participants of the present study, major progress in neonatal intensive care had occurred between the two periods in which they were born (1982–1985 and 1991–1992). The possibility to provide exogenous surfactant stands out as particularly important. Surfactant treatment was unavailable in the early 1980 s, contrasting the 1990 s were surfactant was in regular use; in our unit in the form of Exosurf® as prescribed by the Osiris trial (Group, 1992). Clinically, these changes led to a less severe form of neonatal BPD, often labeled “new BPD” contrasting the “old” and more severe version of the disease (Coalson, 2006) However, despite better treatments the long-term lung function outcomes of the 1982–1985 and 1991−1992 cohorts did not differ significantly, as presented and discussed in detail in a previous communication (Halvorsen et al., 2006). In line with all long-term follow-up studies of preterm born individuals, this present study is faced with the obvious limitation that data cannot necessarily be extrapolated to survivors of today's situation, where more immature individuals exposed to more advanced therapies survive and grow up. Differences in neonatal practices, both between time eras and geographical areas, impact survival rates and long−term outcomes in ways that can be difficult to predict (Kotecha et al., 2013; Vollsaeter et al., 2015). This scenario calls for continuing “surveillance processes” in the form of comprehensive follow-up studies that can provide valuable feedback to involved health care providers. BPD has in many studies been linked to lower FEV_1_, diffusing capacity and peak aerobic exercise capacity (Bader et al., 1987; Santuz et al., 1995; Jacob et al., 1997; Kriemler et al., 2005; Joshi et al., 2013) in childhood and adolescence. In our previous published study (Clemm et al., 2012), we did not find that BPD was related to $\dot{\text{V}}$O_2peak_.

Reduced pulmonary capillary capacitance and pulmonary vascular conductance may have secondary cardiovascular effects with a lower maximal cardiac output and oxygen pulse ($\dot{\text{V}}$O_2_/HR). The slope of the relationship between HR and $\dot{\text{V}}$O_2_ was higher in EP-born group, but the intercept was not different. When extrapolating to the maximal HR, which was the same, $\dot{\text{V}}$O_2max_ will be lower. $\dot{\text{V}}$O_2peak_ was numerically lower in the EP−born participants, but was not significantly different from the term-born control group when adjusted for weight. However, the significantly steeper HR−$\dot{\text{V}}$O_2_ slope supports the numerical data, particularly as other studies have demonstrated a lower $\dot{\text{V}}$O_2peak_ in EP−born children (Kilbride et al., 2003; Smith et al., 2008; Burns et al., 2009; Welsh et al., 2010; Clemm et al., 2012, 2014, 2015).

The oxygen pulse ($\dot{\text{V}}$O_2_/HR) is just a mathematical transformation of the HR-$\dot{\text{V}}$O_2_ relationship, and when related to $\dot{\text{V}}$O_2_ it is a hyperbola with the asymptote, or the maximal $\dot{\text{V}}$O_2_/HR being equal to the slope of the linear relationship. When oxygen extraction or arteriovenous difference in oxygen content is the same, $\dot{\text{V}}$O_2_/HR is related to stroke volume. So, if oxygen extraction is the same, participants born extremely preterm have lower maximal stroke volume. Aerobic capacity is related to habitual physical activity and exercise training, and can also be related to stroke volume. It was shown in the previously published study that the EP-born participants were less active than the term−born, and that the level of physical activity was related to $\dot{\text{V}}$O_2peak_(Clemm et al., 2012). However, the relationships between level of physical activity and $\dot{\text{V}}$O_2peak_ were not different in the two groups.

With respect to the relationship between HR and $\dot{\text{V}}$O_2_ a lower slope is expected in physically fit participants. Only the slope, and not the intercept, was lower in the term−born group, which means that resting heart rate is lower. The intercept is the extreme end of the relationship and has no physiological meaning. Whether differences in physical fitness or cardiovascular effects secondary to the differences in lung function contribute to a lower VO_2peak_ remains unanswered. However, if EP−born participants have exercise induced pulmonary hypertension as indicated in a recent study (Abman et al., 2017), a higher slope of the HR − $\dot{\text{V}}$O_2_ relationship would be expected, and a higher slope of the V_E_ − $\dot{\text{V}}$CO_2_ is a characteristic of pulmonary hypertension (Schwaiblmair et al., 2012). The functional significance is modest only and the alveolar to arterial difference in oxygen pressure appear not to be widened (Duke et al., 2014), indicating that ventilatory capacity is not limiting exercise.

In the majority of the children and adolescents the responses could be described by mathematical models. We have previously shown that this can be done in healthy physically fit young adults (Kjelkenes and Thorsen, 2010) and in patients having chronic obstructive lung disease (Frisk et al., 2014). In healthy participants there were no differences in the curve parameters between maximal and submaximal exercise tests with the same protocol done on separate days within 1 week (Kjelkenes and Thorsen, 2010). Any criteria for having achieved the maximum oxygen uptake are less important when the trajectories of the responses are compared. However, that is not to say that the maximum or peak responses achieved are not important by themselves.

We conclude that the differences observed in ventilatory efficiency between EP and term−born groups were due to a higher alveolar deadspace, and that breathing patterns were similar in the EP and term−born groups after adjusting for lung and body size. The responses to exercise could be satisfactorily described by the mathematical models on an individual basis, providing important physiological information beyond what can be extracted from peak responses.

## Author contributions

There are two first authors; JH and HH; they have contributed equally to the study and writing this manuscript. All authors have contributed substantial to the concept and design of the study. All authors have revised the manuscript critically for important intellectual content and final approved the version to be published. There is an agreement among the authors for all aspect of the work in this study, ensuring that questions related to the accuracy and any part of the work are appropriately investigated and resolved.

### Conflict of interest statement

The authors declare that the research was conducted in the absence of any commercial or financial relationships that could be construed as a potential conflict of interest.
